# Risk Assessment of Arsenic Mitigation Options in Bangladesh

**Published:** 2006-09

**Authors:** Guy Howard, M. Feroze Ahmed, Abu Jafar Shamsuddin, Shamsul Gafur Mahmud, Daniel Deere

**Affiliations:** ^1^ Arsenic Policy Support Unit, 5th Floor, DPHE Bhaban, 14 Shaheed Capt. Monsur Ali Sharani, Kakrail, Dhaka 1000, Bangladesh; ^2^ Bangladesh University of Engineering and Technology, Dhaka 1000; ^3^ International Training Network, Bangladesh University of Engineering and Technology, Dhaka 1000; ^4^ Water Futures, 32 Sirius Street, Dundas Valley, NSW 2117, Australia

**Keywords:** Arsenic, Arsenic mitigation: Water pollution, Water supply, Water quality, Risk assessment, DALYs, Pathogens, Technology choice: Bangladesh

## Abstract

The provision of alternative water sources is the principal arsenic mitigation strategy in Bangladesh, but can lead to risk substitution. A study of arsenic mitigation options was undertaken to assess water quality and sanitary condition and to estimate the burden of disease associated with each technology in disability-adjusted life years (DALYs). Dugwells and pond-sand filters showed heavy microbial contamination in both dry and monsoon seasons, and the estimated burden of disease was high. Rainwater was of good quality in the monsoon but deteriorated in the dry season. Deep tubewells showed microbial contamination in the monsoon but not in the dry season and was the only technology to approach the World Health Organization's reference level of risk of 10^-6^ DALYs. A few dugwells and one pond-sand filter showed arsenic in excess of 50 μg/L. The findings suggest that deep tubewells and rainwater harvesting provide safer water than dugwells and pond-sand filters and should be the preferred options.

## INTRODUCTION

The contamination of shallow groundwater used for water supply with arsenic in Bangladesh represents a significant public-health concern for the country. From the initial detection in 1993, the extent of arsenic contamination has become better characterized and has been described by some as one of the most significant poisoning threats from water worldwide (1). A nationwide survey undertaken in the late 1990s suggested that 27% of all shallow tubewells were likely to have arsenic contamination above the Bangladesh standard of 50 μg/L with 46% in excess of the provisional World Health Organization (WHO) guideline value of 10 μg/L (2). Subsequently, blanket screening of shallow tubewells has been undertaken in 271 affected upazilas, with about five million tubewells having been tested (3). This screening showed that 29% of the tubewells in the affected upazilas had arsenic in excess of the Bangladesh standard.

These results suggest that the overall scale of the problem may, in fact, be less than originally suspected (4). Nonetheless, where arsenic contamination does occur, it represents a potentially serious public-health threat for many ordinary Bangladeshis. The provision of water supplies with acceptable levels of arsenic is the only proven means of reducing the threat to exposed populations.

Progress on mitigation of arsenic risks has lagged behind the definition of the scale and nature of the problem, and provision of mitigation options remains a major challenge. The major emphasis for the mitigation of arsenic in Bangladesh is the provision of alternative water sources. A number of alternative water-supply technologies have been identified and tested on a pilot basis in several areas of Bangladesh. Options include improved dugwells, deep tubewells, pond-sand filters, and rainwater harvesting. In addition, piped water supplies from various sources have been piloted, and work is ongoing to develop improved designs for multi-stage filtration units that can take water from larger water bodies, such as rivers, canals, and baors.

The key policy lesson for public-health protection that emerges from the arsenic crisis in Bangladesh is that, in improving water-supply services, consideration must be given to the degree of public-health risk substitution that may result from the intervention (5). In the case of Bangladesh, the provision of tubewells tapping the shallow aquifer substituted one public-health risk (diarrhoeal disease from microbial pathogens) by another (from arsenic). This risk substitution was not predicted at that time, and the evidence of the potential for such a substitution was certainly not adequate for an evaluation of the probability and nature of potential risk substitutes. In the mitigation of arsenic, however, the potential risk substitution is well-known and can be quantified, albeit within estimable levels of uncertainty.

Understanding risk substitution is critical to technology selection and sustainable arsenic mitigation. In developing a mitigation option, public-health protection would demand that a key criterion for technology selection should be based on the potential public-health consequence of using a particular technology. Although such decisions are normally based on a number of considerations (financial, environmental, social, etc.), as a general principle, the technology representing the least public-health risk would usually be selected. This is consistent with approaches to establishing health-based targets for water supply outlined in the third edition (2004) of the WHO Guidelines for Drinking-water Quality (6).

Risk substitution is a relatively common phenomenon in water supply, and attempts are increasingly made to quantify ‘competing’ risks to inform decision-making. Good examples of risk substitution are the risks associated with disinfection by-products resulting from introducing chlorination and ozonation of water supplies, or the increasing exposure to Legionella as a result of introducing piped water supplies. In the case of disinfection by-products, the use of chemicals to remove pathogenic bacteria leads to a small increase in risk of cancers from chemicals formed during the oxidation reactions but this is justified because of the much greater decrease in risks from microbial pathogens. In the case of *Legionella*, this environmental organism is unlikely to present more than very limited human health risks unless warm water environments are created, such as through piped hot water systems operating at relatively low temperature or through some types of evaporative cooling systems. Therefore, in both the cases, the risk substitutes are generally considered to present much lower risks than those risks that would otherwise be present.

In the context of arsenic mitigation, three principal types of hazard may potentially substitute for arsenic from water supply (5). These are: (a) Microbial hazards: pathogens derived from human and animal faeces that cause diarrhoea and a range of other diseases, some with significant chronic sequelae; (b) Toxins derived from cyanobacteria that may lead to adverse health effects, including liver cancer; and (c) Other chemical contaminants in source water from natural sources or introduced from pollution.

In considering the causes of reported waterborne diseases, microbial hazards are more commonly associated with greater levels of risk than chemical hazards. In developing countries, microbial hazards account for a very significant proportion of the burden of disease. Diseases due to microbial hazards from poor water, sanitation, and hygiene are responsible for an estimated 3.7% of the total global burden of disease (7). In addition, for microbial hazards, as for carcinogenic risks from chemicals, such as arsenic, it is generally assumed that there is no safe threshold since any exposure has the potential to initiate infection.

When comparing the risks associated with arsenic and microbial hazards, several important points emerge. Both are strongly influenced by poverty and nutrition (5, 8). Risks of infection by microbial hazards increase markedly with increasing poverty, and the overall health burden from microbial hazards is significantly greater in poorer communities. Arsenicosis also appears to be related to poverty and has a greater incidence among poorer households exposed to elevated concentrations of arsenic. There is a link between nutritional status and illness from pathogen infection and arsenicosis. For pathogens, this is a two-way process with under-nutrition increasing susceptibility to disease and repeated infections, resulting in continued under-nutrition (5). The situation with arsenic is less clear, but is likely to operate in a similar way (8).

Cyanobacterial blooms occur in some ponds in Bangladesh. The risks associated with toxins derived from cyanobacteria include gastrointestinal diseases from acute exposure and liver cancer from chronic exposure. In addition to direct adverse health effects, algal blooms often lead to significant taste and odour problems. The overall health impact of cyanobacterial toxins remains unclear, but the potential risk substitution is sufficiently large to suggest that only protected surface water sources are used or that the water is clarified prior to filtration. Other potential hazards include chemicals derived from pollution and levels of manganese in groundwater. How-ever, the overall risks associated with such pollution will generally be significantly lower than for microbial hazards and arsenic.

To provide some quantification of the public-health risks associated with the use of alternative water sources, a study was undertaken with support from the Arsenic Policy Support Unit over 12 months on the quality of water, sanitary condition, and social acceptability of water supplies provided as arsenic mitigation. In addition, a quantitative health risk assessment (QHRA) model was developed and used for providing an estimation of disease burden associated with each technology.

## MATERIALS AND METHODS

The four principal alternative water-supply technologies used in arsenic mitigation to date are: dugwells, deep tubewells, pond-sand filters, and rainwater harvesting systems. Dugwells are large diameter wells manually constructed, lined with concrete rings, and covered by a concrete slab or a metal sheet with ventilation. A hand-pump is used for withdrawing the water. A small apron to protect against short-circuiting by contaminants sur-rounds dugwells. The deep tubewells in the study all had hand-pumps for water withdrawal and were sunk into deeper aquifers (typically 300 feet or more). All the deep tubewells had small aprons around the wellhead to provide protection against short-circuiting by contaminants. The pond-sand filters are designed as a slow sand filtration system with water drawn from an adjacent pond. In this study, the pond-sand filters had an initial roughing filter to reduce turbidity and a final sand-bed for slow sand filtration. The rainwater harvesting systems were all individual household systems with foul-flush mechanisms and taps for removal of water. The designs for each technology were consistent within each group. Although the water supplies were constructed under different programmes, in many cases, the construction agency did not vary. The study did not attempt to analyze performance by mitigation programme, as this would have required additional survey design criteria and was not the primary interest of the study.

In all cases, ongoing operation and management of the mitigation options was the responsibility of the community, and caretakers had been identified and trained for all water sources visited, although their activity level varied. Assessment of the performance of operation and maintenance was undertaken through sanitary inspection, and a social assessment was performed at the same time.

Water sources included in the study were selected using a modified cluster survey approach, with the confidence level set at 0.95, power set at 0.8, precision at 0.1, and a design effect of 2 (9). Two sets of water sources were selected. The first set were dugwells and deep tubewells installed as arsenic mitigation options. For this set, 72 water supplies were calculated as being required for statistical representivity. In addition, sampling of 24 shallow tubewells was undertaken in the dry season to provide a comparison in quality of water and health risk. The second set were pond-sand filters and rainwater harvesting systems, for which it was calculated that 84 water sources were required. For both the sets, the total water sources were divided into equal numbers of each water source type. This resulted in 36 dugwells and 36 deep tubewells, and 42 pond-sand filters and 42 rainwater harvesting being sampled.

Based on a review of data on water supplies installed across the country, the dugwells and deep tubewells were divided into six clusters each and the pond-sand filters and rainwater harvesting into seven clusters each. Clusters were randomly selected across the country using a proportional weighting table. Samples for water-quality analysis were taken in both dry and monsoon seasons from all sources during 2004–2005.

All water sources were tested for thermotolerant coliforms as the principal indicator of faecal contamination in line with recommendations of the WHO (6). The thermotolerant coliforms were analyzed using a portable field test-kit employing membrane filtration and membrane lauryl sulphate broth. The plates were incubated for 14–18 hours, and yellow colonies were counted as thermotolerant coliforms. Confirmatory testing of *Escherichia coli* was undertaken in the laboratory of Bangladesh University of Engineering and Technology (BUET) using the multiple tube method. Analysis of arsenic was undertaken by atomic adsorption spectro-photometer in the BUET laboratory. All other chemical analyses were performed using a HACH spectrophoto-meter in the BUET laboratory. The existing quality-control procedures of BUET laboratory were rigorously followed in all analyses.

Sanitary inspections were carried out on all water supplies using a standardized format (10). Questionnaires with a mixture of observations and community interview questions were prepared based on existing examples and filled in for each water source at the time of sampling.

A range of physical and chemical parameters was tested for each technology, although only arsenic data are reported here, as this was the only chemical parameter for which a likely disease-burden estimate was derived.

The data for thermotolerant coliforms and arsenic were used for estimating the likely burden of disease using disability-adjusted life years (DALYs), which is the globally applied metric used for comparing different disorders and diseases with different health outcomes. The model structure and assumptions were described elsewhere (9) and are only summarized here.

The WHO recommends the use of reference pathogens when undertaking a quantitative assessment of microbial risk (6). A reference pathogen is one whose characteristics (infectivity, burden of disease, and ubiquity) would mean that its control would have controlled the risks from all similar pathogens (11). This means that detailed risk assessments for each individual pathogen that could be present, which would be difficult (because of limited data) or expensive, are avoided. In this study, three reference pathogens were selected. A generalized pathogenic *E. coli* was developed drawing on characteristics of *Salmonella* ubiquity, *Shigella dysenteriae* infectivity, and a disease burden from *E. coli* O157:H7 to act as a reference for bacterial pathogens. Rotavirus was used to act as a reference for viral pathogens and *Cryptosporidium parvum* to act as a reference for protozoan pathogens. Data for unboiled water consumption was taken from recent literature (12) and dose-responses were selected from published literature (13–16), and well-validated models were applied. Only the effect of diarrhoea (morbidity and mortality) was taken into account, given the limited data on sequelae of infection in Bangladesh. The degree of acquired immunity to these pathogens was built into the model taking into account local data on infection patterns and other routes of transmission.

No direct assessment of presence of pathogens was made in the water samples and, therefore, assumptions were made regarding the relationship between pathogens and indicator bacteria. Ratios were derived from long-term monitoring of pathogens and *E. coli* in sewage from two large studies in Australia. In the absence of local data, these were considered to be sufficiently reliable for use in the assessment.

Arsenic end-points considered were early onset of symptoms (keratosis and melanosis), skin cancer, lung cancer, and bladder cancer as these were the end-points for which reliable data were available. Other effects of kidney, liver, and prostate cancer and cardiovascular, endo-crine, reproductive and cognitive effects were not included as less conclusive evidence is currently available (17–19). The arsenic dose-response models of Yu *et al*. (16) were used for the cancer end-points included. An initial attempt was made to use the West Bengal data of Mazumder *et al*. (20) to predict early onset of symptom development, but these were subsequently omitted because there is no agreement on severity weights to be applied for these symptoms. Life expectancy was based on the estimated average life expectancy in Bangladesh in 2004 of 62 (which was based on an average life expectancy of 60 years in 1999 and likely improvement since then).

## RESULTS

**Fig. 1.**
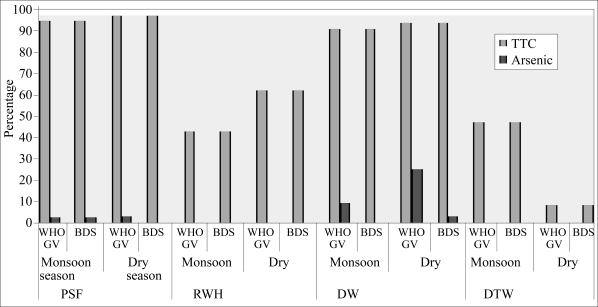
Quality of water in relation to the Bangladesh standard for TTCs and arsenic

The results of the testing of thermotolerant coliforms and arsenic are shown for each technology in the dry and wet seasons in Table 1. Figure 1 shows the percentage of samples in the wet and dry seasons that exceeded the Bangladesh standards for each parameter.

**Table 1.** Water-quality results for technology in dry and monsoon seasons

Parameter		Dugwell		Deep tubewell		Pond-sand filter		Rainwater harvesting	
		Dry season	Monsoon season	Dry season	Monsoon season	Dry season	Monsoon season	Dry season	Monsoon season
TTC cfu/100 mL	Minimum	<1	<1	<1	<1	<1	<1	<1	<1
	Medium	47	820	1	<1	37	107	2	<1
	Mean	163	1,998	<1	10.5	91	255	14	43.9
	Maximum	TNTC	1,5000	27	160	590	2,200	122	640
Arsenic μg/L	Minimum	0.00	0.01	0.00	0.05	1.00	0.50	0.00	0.00
	Medium	0.79	0.60	0.41	0.10	3.00	0.50	0.00	0.00
	Mean	8.14	3.70	1.05	1.30	3.57	3.02	0.55	0.55
	Maximum	108.00	25.00	8.95	6.50	11.00	65.5	6.00	6.00

TNTC=Too numerous to count;

TTC=Testing of thermotolerant coliform

BDS=Bangladesh standard;
DTW=Deep tubewell;
DW=Dugwell;
GV=Guideline value;
PSF=Pond-sand filter;
RWH=Rainwater harvesting;
TTCs=Thermotolerant coliforms;
WHO=World Health Organization

The data revealed that all the alternative water sources showed some degree of microbial contamination on, at least, a seasonal basis. The dugwells and pond-sand filters showed the most regular and intense microbial contamination. Even in the dry season, when contamination would be expected to be lowest, over 90% of samples from these technologies exceeded the Bangladesh standard for thermotolerant coliforms. Average and median concentrations for both the technologies were very high in the monsoon season and remained relatively high in the dry season. However, both the technologies had a minimum count of <1 cfu/100 mL in both the seasons showing that good microbial quality can be assured.

The deep tubewells and rainwater harvesting had better microbial quality, but the rainwater harvesting showed regular contamination, rising to 60% exceeding the Bangladesh standard in the dry season. Even the deep tubewells, which, in the dry season, showed very low contamination (8%), increased to almost 50% in the monsoon season. The intensity of contamination was much lower in general than the pond-sand filters and dugwells.

The WHO Guidelines for Drinking-water Quality do not have a precise value for microbial quality, rather have guidelines for verification under a water-safety plan (6). For these supplies covered in this assessment, which typically serve less than 5,000 population each, the WHO (6) recommends that the following grading scheme be used:

90% and above samples negative for TTC or *E. coli*=Excellent

80 to 90% samples negative for TTC or *E. coli*=Good

70 to 80% samples negative for TTC or *E. coli*=Fair

60 to 70% samples negative for TTC or *E. coli*=Poor

Using this grading scheme, the dugwells, pond-sand filters, and rainwater harvesting would be considered in the poor category. The deep tubewells would be considered of excellent quality in the dry season, but poor quality in the monsoon season, with an overall rating of good quality.

The sanitary inspection data are presented in Table 2, and this shows that the dugwells and pond-sand filters had high sanitary risks, whereas sanitary risks in rainwater harvesting were much lower.

**Table 2.** Sanitary inspection data for the options included in the assessment

Technology	Minimum sanitary inspection score (%)	Median sanitary inspection score (%)	Maximum sanitary inspection score (%)
Dugwell	2	4	8
Deep tubewell	2	4	7
Pond-sand filter	1	4	6
Rainwater harvesting	0	1	5

The deep tubewells also had relatively high sanitary risks, but the levels of contamination suggest that the technology is more robust in terms of preventing microbial contamination.

**Fig. 2.**
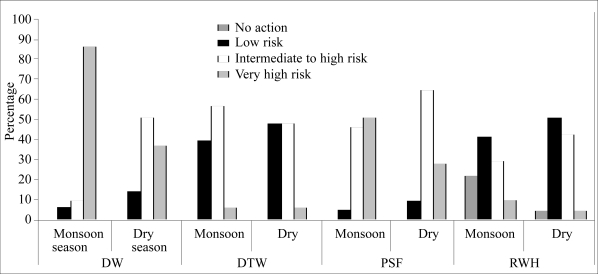
Combined risk assessment for microbial contamination

Combined analysis was performed on the thermotolerant coliform and sanitary inspection data as undertaken following the process outlined by Lloyd and Bartram (21) and WHO (6). This analysis is shown in Fig. 2.

DTW=Deep tubewell;
DW=Dugwell;
PSF=Pond-sand filter;
RWH=Rainwater harvesting

This analysis demonstrated the seasonal nature of risk, although the season of the greatest risk was the dry season for rainwater harvesting and monsoon season for the other technologies. Overall, the deep tubewells and rainwater harvesting had lower risk ratings compared to the other technologies, although significant numbers were at an intermediate risk.

The pond-sand filters had a generally lower risk profile than the dugwells, but significant numbers were at very high risk even in the dry season. These data showed that the majority of dugwells were in the intermediate/high and very risk categories in both monsoon and dry seasons, with an alarming number in the very high-risk category in the monsoon season.

The arsenic data showed that, in general, most technologies provided water with low arsenic concentrations. As expected, rainwater harvesting had the least arsenic. Both dugwells and pond-sand filters had some sources with arsenic above the Bangladesh standard, and a higher number of dugwells had arsenic above the provisional WHO guideline value of 10 μg/L. The arsenic in the pond-sand filters was probably due to recharging of the pond with water from a contaminated tubewell. Arsenic decreased in the dugwells in the monsoon, probably because of dilution in the very shallow aquifer.

**Fig. 3.**
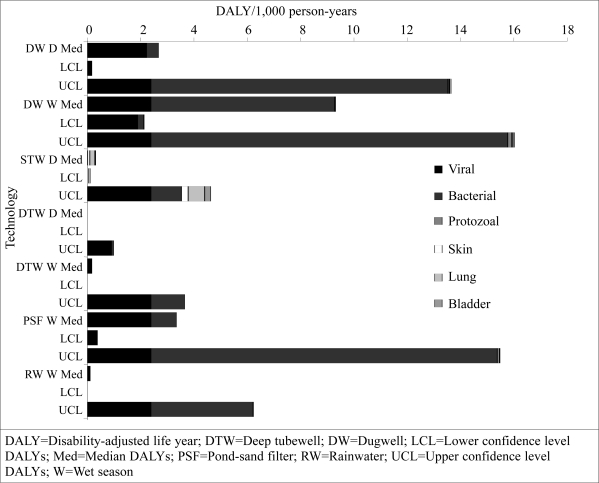
Burden of disease by technology and season

Figure 3 shows the outcome of the disease-burden estimate for each technology by season. These data showed that the burden of disease derived from pathogens was greater than that from arsenic and that viral risks were most dominant at lower concentrations of indicator organisms. The disease-burden estimates increased significantly for dugwells and pond-sand filters in the monsoon and to a lesser extent for deep tubewells. The disease-burden estimates increased for rainwater harvesting in the dry season. To determine the level of risk in comparison to the WHO reference level of risk of 10^-6^ DALYs (6), comparisons were made with the upper 95% confidence interval on the burden of disease from bacterial pathogens because bacterial pathogens dominated the burden of disease. The findings are summarized in Table 4.

DALY=Disability-adjusted life year;
DTW=Deep tubewell;
DW=Dugwell;
LCL=Lower confidence level DALYs;
Med=Median DALYs;
PSF=Pond-sand filter;
RW=Rainwater;
UCL=Upper confidence level DALYs;
W=Wet season

**Table 4.** Summary of upper 95% confidence limits of the risk estimate expressed as DALYs per person per year. The WHO reference level of risk is 1×10^-6^

Technology	Dry season	Monsoon season
Dugwell	1.11×10^-2^	1.34×10^-2^
Deep tubewell	6.98×10^-5^	1.26×10^-3^
Pond-sand filter	1.07×10^-2^	1.30×10^-2^
Rainwater harvesting	6.48×10^-3^	3.83×10^-3^

DALY=Disability-adjusted life year;

WHO=World Health Organization

The upper bounds of the disease-burden estimates for dugwells and pond-sand filters were at around four orders of magnitude greater than the WHO reference level of risk (10^-6^) in the monsoon season and the dry season. In the monsoon season, the upper bounds of the disease-burden estimates for deep tubewells and rainwater harvesting were around one order of magnitude less than pond-sand filters and dugwells but were still around three orders of magnitude greater than the reference level of risk. During the dry season, the upper-bound risk estimates for rainwater harvesting were similar to (slightly higher than) those estimated for the monsoon season, whereas the upper-bound estimates for deep tubewells in the dry season were almost down at the reference level of risk.

For the arsenic DALYs, the dugwells, deep tubewells, and pond-sand filters had associated upper risk estimates only slightly above the WHO reference levels of risk in the dry and wet seasons, whereas rainwater harvesting had associated upper risk estimates below the WHO reference level of risk.

## DISCUSSION

The results showed that the microbial quality of water supplied from all types of water sources was prone to deterioration and that the public-health risks were predicted to increase as a result. Therefore, the delivery of safe drinking-water continues to represent a significant priority in rural areas of Bangladesh. Data collected in the dry season under this study also showed that shallow tubewells were commonly contaminated and had a higher level of contamination than deep tubewells, although lower than dugwells.

The deterioration in microbial quality during the monsoon season was most pronounced for dugwells and pond-sand filters. The results suggest that, in many cases, the designs used were not adequate to provide public-health protection, and maintenance was poor. In the latter cases, this clearly suggests that communities had not received sufficient ongoing support in maintaining their water supplies. These findings for dug wells are similar to those reported by other organizations (22–25).

The authors are aware of other data for dugwells showing a relatively better performance that have been collected by the Dhaka Community Hospital, although these only covered the dry season. Furthermore, subsequent studies (that are yet unpublished) in Jessore district by United Nations Children's Fund and Japan International Cooperation Agency have found a better performance of dugwells with an improved design and chlorination, although again the data are restricted to dry season sampling. This suggests that it is possible to improve performance of dugwells and that these remain a viable option in areas where communities prefer these to other options and where providers are able to offer post-construction support. However, if this approach is followed, more information is required on overall costs of both hardware and software.

The pond-sand filters continue to perform poorly and, although no estimate was made of the burden of disease associated with cyanobacterial toxins, ponds frequently showed algal blooms, and it is unlikely that the technology currently used would provide any significant removal. Chlorination of pond-sand filters will improve the quality, but the sustainability of this approach is question-able and the benefits in terms of cyanotoxins are likely to be minimal.

The deep tubewells and rainwater harvesting appeared to offer the best-quality water and the lowest overall burden of disease. However, both the technologies did show microbial contamination, and again, this reflects poor maintenance, and by implication, inadequate support and training of communities. Contamination of the deep tubewells increased in the monsoon season and was almost certainly due to use of contaminated priming water.

The quality of rainwater harvesting deteriorated in the dry season, and the disease-burden estimate also increased a little. The mechanism of contamination of rainwater tanks is not certain, but may be either due to re-growth of thermotolerant coliform bacteria within the tanks or because the water sampled was derived from rain from one of the occasional storms that occur in the pre-monsoon period. If the contamination results from the former process, the health risk may be lower than predicted from the disease-burden tool. A review by Hunter of the evidence of gastrointestinal diseases derived from heterotrophic bacteria that re-grow in water supplies showed that there is no credible epidemiological evidence of a link between re-growth and gastrointestinal diseases (26). It is quite possible that the thermotolerant coliforms found in the dry season are environmental organisms and of limited public-health significance. If contamination is due to recent rainfall, it demonstrates that households have not taken up the recommended practice of first-flush diversion and have allowed contaminated water to enter the tanks. The later improvement during the monsoon season would then reflect that faecal material does not build up on the catchment.

For most samples, none of the mitigation options showed significant arsenic contamination, and all options can be considered to be effective in reducing arsenic exposure. A study by the Asia Arsenic Network has shown significant arsenic contamination of dugwells in Jessore, when 46% of 51 dugwells tested, had arsenic in excess of the Bangladesh standard (23). Likewise, the Asia Arsenic Network found that 10% of deep tubewells also had arsenic in excess of the Bangladesh standard (24). In the BGS-DPHE national study in 1999, 1% of deep tubewells across the country were found to have raised arsenic (2). Although, in our study, the deep tubewells had low arsenic, there remain concerns about their unrestricted use in the absence of reliable data on the location of the Pleistocene aquifer and an overlying aquiclude.

The disease-estimation tool used provided a useful means of comparing different technologies, but, as in many risk-assessment methodologies, relies heavily on the assumptions made. Sensitivity analysis showed that the model was sensitive to the assumed relationships between thermotolerant coliforms and *E. coli*, infectivity of pathogenic *E. coli*, the volume of unboiled water consumed, and immunity to rotavirus. These are all issues that would benefit from further research.

Whether the choice of rotavirus as a reference pathogen in Bangladesh is correct is an area of significant discussion, given its strong relationship to hygiene as opposed to water. Hepatitis E virus may offer a better means of assessing viral risks, particularly as it is a common pathogen in Bangladesh and linked to regular outbreaks. The lack of data on sequelae is a further issue of concern, as this may under-estimate overall burdens of disease, particularly for bacterial pathogens. However, the exclusion of sequelae for bacteria can be justified in the light of limited data on the impact of immune status due to HIV status and poor nutrition that may increase the burden of disease from viral and protozoan pathogens (27).

In terms of the outcomes from the upper disease-burden estimates, only the deep tubewells in the dry season approach the WHO reference level of risk. The other supplies showed the upper disease-burden estimates to be significantly higher than this level of risk. Furthermore, the use of all these supplies entails trans-port of water to home and, thus, re-contamination is a common problem. This may result in a significantly raised risk at the point of consumption, which has been found in similar settings (27).

All the water supplies offered as arsenic mitigation solutions showed significant problems with microbial quality. By undertaking the disease-burden estimates, it can be shown that none of the supplies can be said to deliver ‘safe’ drinking-water under all circumstances as they are currently employed. On the other hand, all the technologies had low arsenic showing that all are effective at least in reducing arsenic exposure. Furthermore, under good situational and operational conditions, all can prove effective at reducing burdens of microbial diseases.

Looking at the body of evidence, overall, deep tubewells and rainwater harvesting appear to offer the lowest public-health risk, although neither is free of problems. The development of deep tubewells is also constrained by the limited information on the Pleistocene aquifer and its sustainable development. The dugwells and pond-sand filters were found in this study to perform particularly poorly and have both high sanitary risks and heavy microbial contamination, particularly in the monsoon season. The continued use of these technologies requires that designs be improved and that agencies provide more training and better support to communities in operation and maintenance.

The use of tools to estimate burdens of disease associated with different technologies has proved to be valuable in supporting technology choice. This tool would benefit from further development, but already has proven to be robust enough to assist decision-making. The advantage of using such tools is that they reinforce the public-health focus of water-supply provision and can be used for assessing the degree to which interventions that improve water supplies will reduce public-health risks.

## ACKNOWLEDGEMENTS

The authors thank the Department for International Development (DFID), UK for their support in funding the research leading to this paper. The views expressed in this paper are those of the authors and do not reflect the official position of DFID or the Government of Bangladesh.

Work is to be attributed to: Arsenic Policy Support Unit and International Training Network Centre, Dhaka.
